# (Di-2-pyridyl­amine-κ^2^ *N* ^2^,*N* ^2′^)diiodidoplatinum(II)

**DOI:** 10.1107/S1600536812011907

**Published:** 2012-03-24

**Authors:** Kwang Ha

**Affiliations:** a School of Applied Chemical Engineering, The Research Institute of Catalysis, Chonnam National University, Gwangju 500-757, Republic of Korea

## Abstract

The Pt^II^ ion in the title complex, [PtI_2_(C_10_H_9_N_3_)], is four-coordinated in a distorted square-planar environment defined by the two pyridine N atoms of the chelating di-2-pyridyl­amine (dpa) ligand and by two I^−^ anions. The dpa ligand is not planar, the dihedral angle between the pyridine rings being 52.8 (3)°. Pairs of complex mol­ecules are assembled through inter­molecular N—H⋯I hydrogen bonds, forming a dimer-type species. The complexes are stacked in columns along the *b* axis and display several inter­molecular π–π inter­actions between the pyridine rings, with a shortest ring centroid–centroid distance of 3.997 (5) Å.

## Related literature

For the crystal structure of the related chlorido Pt^II^ complex [PtCl_2_(dpa)], see: Li & Liu (2004 ▶); Tu *et al.* (2004 ▶); Zhang *et al.* (2006 ▶).
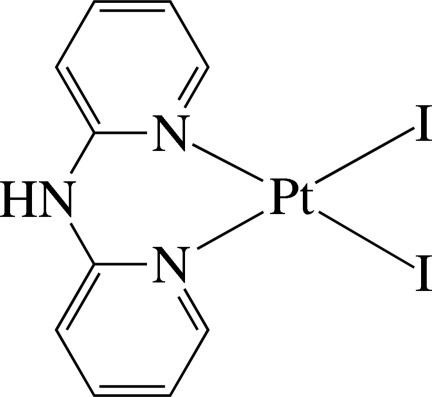


## Experimental

### 

#### Crystal data

[PtI_2_(C_10_H_9_N_3_)]
*M* *_r_* = 620.09
Monoclinic, 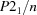

*a* = 8.2354 (6) Å
*b* = 9.7940 (7) Å
*c* = 16.4702 (12) Å
β = 102.148 (1)°
*V* = 1298.70 (16) Å^3^
*Z* = 4
Mo *K*α radiation
μ = 15.54 mm^−1^
*T* = 200 K
0.16 × 0.12 × 0.08 mm

#### Data collection

Bruker SMART 1000 CCD diffractometer
Absorption correction: multi-scan (*SADABS*; Bruker, 2000 ▶) *T*_min_ = 0.753, *T*_max_ = 1.000
7763 measured reflections
2527 independent reflections
2206 reflections with *I* > 2σ(*I*)
*R*_int_ = 0.028

#### Refinement

*R*[*F* ^2^ > 2σ(*F* ^2^)] = 0.031
*wR*(*F* ^2^) = 0.077
*S* = 1.07
2527 reflections
145 parameters
H-atom parameters constrained
Δρ_max_ = 1.47 e Å^−3^
Δρ_min_ = −1.31 e Å^−3^

### 

Data collection: *SMART* (Bruker, 2000 ▶); cell refinement: *SAINT* (Bruker, 2000 ▶); data reduction: *SAINT*; program(s) used to solve structure: *SHELXS97* (Sheldrick, 2008 ▶); program(s) used to refine structure: *SHELXL97* (Sheldrick, 2008 ▶); molecular graphics: *ORTEP-3* (Farrugia, 1997 ▶) and *PLATON* (Spek, 2009 ▶); software used to prepare material for publication: *SHELXL97*.

## Supplementary Material

Crystal structure: contains datablock(s) global, I. DOI: 10.1107/S1600536812011907/wm2603sup1.cif

Structure factors: contains datablock(s) I. DOI: 10.1107/S1600536812011907/wm2603Isup2.hkl

Additional supplementary materials:  crystallographic information; 3D view; checkCIF report

## Figures and Tables

**Table:** 

Pt1—N1	2.033 (7)
Pt1—N3	2.055 (6)
Pt1—I2	2.5675 (7)
Pt1—I1	2.5934 (7)

**Table:** 

N1—Pt1—N3	85.9 (3)
I2—Pt1—I1	90.85 (2)

**Table 2:** Hydrogen-bond geometry (Å, °)

*D*—H⋯*A*	*D*—H	H⋯*A*	*D*⋯*A*	*D*—H⋯*A*
N2—H2*N*⋯I1^i^	0.92	2.82	3.607 (7)	144

Symmetry code: (i) 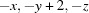.

**Fig. 1.**
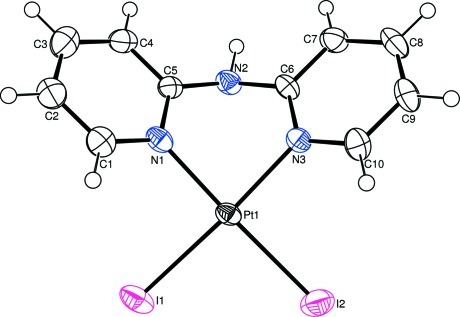
The molecular structure of the title complex, with displacement ellipsoids drawn at the 50% probability level for all non-H atoms.

**Fig. 2.**
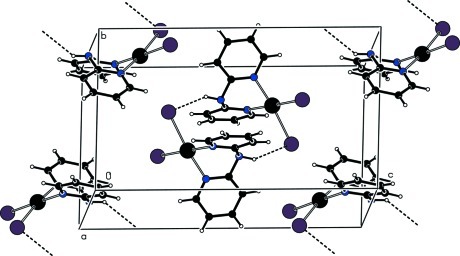
A view of the unit-cell content of the title complex. Intermolecular N—H···I hydrogen-bonding interactions are drawn with dashed lines.

## supplementary crystallographic information

### Comment

The title complex, [PtI_2_(dpa)] (dpa = di-2-pyridylamine, C_10_H_9_N_3_), is closely related with the previously reported analogous chlorido Pt^II^ complex [PtCl_2_(dpa)] (Li & Liu, 2004; Tu *et al.*, 2004; Zhang *et al.*, 2006). The Pt^II^ ion is four-coordinated in a distorted square-planar environment by the two pyridine N atoms of the chelating dpa ligand and two I^-^ anions (Fig. 1). In the crystal, the dpa ligand is not planar. The dihedral angle between the least-squares planes of the pyridine rings is 52.8 (3)°. The two Pt—N and the two Pt—I bond lengths, respectively, are nearly equivalent (Table 1). Two complex molecules are assembled through intermolecular N—H···I hydrogen bonds, forming a dimer-type species (Fig. 2 and Table 2). The complexes are stacked in columns along the *b* axis and display several intermolecular π—π interactions between the pyridine rings, with a shortest ring centroid to centroid distance of 3.997 (5) Å.

### Experimental

To a solution of K_2_PtCl_4_ (0.2082 g, 0.502 mmol) in H_2_O (20 ml) and MeOH (10 ml) were added KI (0.7022 g, 4.230 mmol) and di-2-pyridylamine (0.0896 g, 0.523 mmol) and stirred for 3 h at room temperature. The formed precipitate was separated by filtration and washed with H_2_O and MeOH, and dried at 373 K, to give a yellow powder (0.2614 g). Crystals suitable for X-ray analysis were obtained by slow evaporation from a CH_3_CN/acetone solution.

### Refinement

Carbon-bound H atoms were positioned geometrically and allowed to ride on their respective parent atoms [C—H = 0.95 Å and *U*_iso_(H) = 1.2*U*_eq_(C)]. The nitrogen-bound H atom was located from Fourier difference maps and then allowed to ride on its parent atom in the final cycles of refinement with N—H = 0.92 Å and *U*_iso_(H) = 1.5 *U*_eq_(N). The highest peak (1.47 e Å^-3^) and the deepest hole (-1.31 e Å^-3^) in the difference Fourier map are located 0.56 Å and 0.67 Å from the atoms Pt1 and I1, respectively.

### Crystal data

**Table:**

[PtI_2_(C_10_H_9_N_3_)]	*F*(000) = 1096
*M**_r_* = 620.09	*D*_x_ = 3.171 Mg m^−^^3^
Monoclinic, *P*2_1_/*n*	Mo *K*α radiation, λ = 0.71073 Å
Hall symbol: -P 2yn	Cell parameters from 4677 reflections
*a* = 8.2354 (6) Å	θ = 2.4–26.0°
*b* = 9.7940 (7) Å	µ = 15.54 mm^−^^1^
*c* = 16.4702 (12) Å	*T* = 200 K
β = 102.148 (1)°	Block, yellow
*V* = 1298.70 (16) Å^3^	0.16 × 0.12 × 0.08 mm
*Z* = 4	

### Data collection

**Table:**

Bruker SMART 1000 CCD diffractometer	2527 independent reflections
Radiation source: fine-focus sealed tube	2206 reflections with *I* > 2σ(*I*)
Graphite monochromator	*R*_int_ = 0.028
φ and ω scans	θ_max_ = 26.0°, θ_min_ = 2.4°
Absorption correction: multi-scan (*SADABS*; Bruker, 2000)	*h* = −10→9
*T*_min_ = 0.753, *T*_max_ = 1.000	*k* = −12→9
7763 measured reflections	*l* = −20→20

### Refinement

**Table:**

Refinement on *F*^2^	Primary atom site location: structure-invariant direct methods
Least-squares matrix: full	Secondary atom site location: difference Fourier map
*R*[*F*^2^ > 2σ(*F*^2^)] = 0.031	Hydrogen site location: inferred from neighbouring sites
*wR*(*F*^2^) = 0.077	H-atom parameters constrained
*S* = 1.07	*w* = 1/[σ^2^(*F*_o_^2^) + (0.0328*P*)^2^ + 7.7728*P*] where *P* = (*F*_o_^2^ + 2*F*_c_^2^)/3
2527 reflections	(Δ/σ)_max_ < 0.001
145 parameters	Δρ_max_ = 1.47 e Å^−^^3^
0 restraints	Δρ_min_ = −1.31 e Å^−^^3^

### Special details

**Table:**

Geometry. All e.s.d.'s (except the e.s.d. in the dihedral angle between two l.s. planes) are estimated using the full covariance matrix. The cell e.s.d.'s are taken into account individually in the estimation of e.s.d.'s in distances, angles and torsion angles; correlations between e.s.d.'s in cell parameters are only used when they are defined by crystal symmetry. An approximate (isotropic) treatment of cell e.s.d.'s is used for estimating e.s.d.'s involving l.s. planes.
Refinement. Refinement of *F*^2^ against ALL reflections. The weighted *R*-factor *wR* and goodness of fit *S* are based on *F*^2^, conventional *R*-factors *R* are based on *F*, with *F* set to zero for negative *F*^2^. The threshold expression of *F*^2^ > σ(*F*^2^) is used only for calculating *R*-factors(gt) *etc*. and is not relevant to the choice of reflections for refinement. *R*-factors based on *F*^2^ are statistically about twice as large as those based on *F*, and *R*- factors based on ALL data will be even larger.

### Fractional atomic coordinates and isotropic or equivalent isotropic displacement parameters (Å^2^)

**Table:**

	*x*	*y*	*z*	*U*_iso_*/*U*_eq_	
Pt1	0.06711 (4)	0.84628 (3)	0.151994 (17)	0.02326 (11)	
I1	0.24006 (8)	1.04400 (6)	0.23202 (3)	0.04146 (18)	
I2	−0.14376 (8)	0.86265 (7)	0.24634 (4)	0.04368 (18)	
N1	0.2236 (8)	0.8338 (7)	0.0719 (4)	0.0272 (15)	
N2	−0.0138 (9)	0.8279 (7)	−0.0353 (4)	0.0289 (15)	
H2N	−0.0582	0.8189	−0.0911	0.043*	
N3	−0.0752 (8)	0.7046 (7)	0.0769 (4)	0.0239 (14)	
C1	0.3928 (11)	0.8266 (8)	0.0968 (6)	0.0326 (19)	
H1	0.4403	0.8184	0.1543	0.039*	
C2	0.4957 (11)	0.8309 (9)	0.0416 (6)	0.035 (2)	
H2	0.6126	0.8244	0.0605	0.042*	
C3	0.4278 (12)	0.8447 (8)	−0.0417 (6)	0.038 (2)	
H3	0.4979	0.8505	−0.0806	0.046*	
C4	0.2564 (11)	0.8501 (8)	−0.0688 (5)	0.0313 (19)	
H4	0.2077	0.8616	−0.1260	0.038*	
C5	0.1575 (10)	0.8384 (8)	−0.0103 (5)	0.0242 (17)	
C6	−0.0991 (9)	0.7231 (8)	−0.0054 (5)	0.0232 (16)	
C7	−0.2111 (11)	0.6449 (9)	−0.0613 (5)	0.035 (2)	
H7	−0.2327	0.6645	−0.1191	0.041*	
C8	−0.2908 (11)	0.5374 (9)	−0.0309 (6)	0.037 (2)	
H8	−0.3666	0.4810	−0.0677	0.044*	
C9	−0.2582 (11)	0.5140 (9)	0.0534 (6)	0.038 (2)	
H9	−0.3101	0.4400	0.0753	0.046*	
C10	−0.1502 (10)	0.5980 (9)	0.1058 (6)	0.0315 (19)	
H10	−0.1278	0.5807	0.1638	0.038*	

### Atomic displacement parameters (Å^2^)

**Table:**

	*U*^11^	*U*^22^	*U*^33^	*U*^12^	*U*^13^	*U*^23^
Pt1	0.02847 (18)	0.02197 (18)	0.01700 (16)	−0.00104 (12)	−0.00054 (12)	0.00125 (12)
I1	0.0546 (4)	0.0355 (3)	0.0275 (3)	−0.0086 (3)	−0.0067 (3)	−0.0009 (3)
I2	0.0488 (4)	0.0540 (4)	0.0301 (3)	0.0015 (3)	0.0125 (3)	−0.0015 (3)
N1	0.031 (4)	0.023 (4)	0.024 (3)	−0.001 (3)	−0.001 (3)	0.001 (3)
N2	0.033 (4)	0.031 (4)	0.020 (3)	−0.004 (3)	−0.001 (3)	0.001 (3)
N3	0.023 (3)	0.023 (3)	0.024 (3)	0.003 (3)	0.002 (3)	0.002 (3)
C1	0.034 (5)	0.026 (4)	0.036 (5)	−0.002 (4)	0.002 (4)	0.000 (4)
C2	0.030 (5)	0.033 (5)	0.043 (5)	0.000 (4)	0.006 (4)	−0.002 (4)
C3	0.043 (5)	0.023 (5)	0.053 (6)	0.000 (4)	0.021 (5)	0.003 (4)
C4	0.039 (5)	0.030 (5)	0.026 (4)	0.000 (4)	0.011 (4)	−0.002 (4)
C5	0.034 (4)	0.017 (4)	0.023 (4)	−0.001 (3)	0.007 (3)	−0.002 (3)
C6	0.025 (4)	0.016 (4)	0.026 (4)	0.002 (3)	0.000 (3)	0.001 (3)
C7	0.036 (5)	0.039 (5)	0.027 (4)	−0.002 (4)	0.003 (4)	−0.004 (4)
C8	0.033 (5)	0.026 (5)	0.048 (6)	−0.005 (4)	0.000 (4)	−0.012 (4)
C9	0.042 (5)	0.023 (4)	0.048 (6)	−0.011 (4)	0.008 (4)	−0.005 (4)
C10	0.035 (5)	0.023 (4)	0.036 (5)	0.000 (4)	0.005 (4)	0.006 (4)

### Geometric parameters (Å, º)

**Table:**

Pt1—N1	2.033 (7)	C2—H2	0.9500
Pt1—N3	2.055 (6)	C3—C4	1.390 (13)
Pt1—I2	2.5675 (7)	C3—H3	0.9500
Pt1—I1	2.5934 (7)	C4—C5	1.391 (12)
N1—C5	1.349 (10)	C4—H4	0.9500
N1—C1	1.369 (11)	C6—C7	1.389 (11)
N2—C5	1.388 (10)	C7—C8	1.388 (13)
N2—C6	1.390 (10)	C7—H7	0.9500
N2—H2N	0.9196	C8—C9	1.377 (13)
N3—C6	1.340 (10)	C8—H8	0.9500
N3—C10	1.349 (10)	C9—C10	1.375 (12)
C1—C2	1.368 (13)	C9—H9	0.9500
C1—H1	0.9500	C10—H10	0.9500
C2—C3	1.374 (13)		
			
N1—Pt1—N3	85.9 (3)	C4—C3—H3	120.1
N1—Pt1—I2	176.88 (18)	C3—C4—C5	118.6 (8)
N3—Pt1—I2	91.94 (18)	C3—C4—H4	120.7
N1—Pt1—I1	91.11 (18)	C5—C4—H4	120.7
N3—Pt1—I1	173.33 (18)	N1—C5—N2	117.7 (7)
I2—Pt1—I1	90.85 (2)	N1—C5—C4	121.7 (8)
C5—N1—C1	118.2 (7)	N2—C5—C4	120.5 (7)
C5—N1—Pt1	118.2 (5)	N3—C6—C7	122.1 (7)
C1—N1—Pt1	123.6 (6)	N3—C6—N2	118.7 (7)
C5—N2—C6	120.5 (6)	C7—C6—N2	119.1 (7)
C5—N2—H2N	117.9	C8—C7—C6	118.5 (8)
C6—N2—H2N	99.2	C8—C7—H7	120.7
C6—N3—C10	118.8 (7)	C6—C7—H7	120.7
C6—N3—Pt1	117.5 (5)	C9—C8—C7	118.9 (8)
C10—N3—Pt1	123.7 (6)	C9—C8—H8	120.6
C2—C1—N1	122.2 (8)	C7—C8—H8	120.6
C2—C1—H1	118.9	C10—C9—C8	119.8 (8)
N1—C1—H1	118.9	C10—C9—H9	120.1
C1—C2—C3	119.2 (8)	C8—C9—H9	120.1
C1—C2—H2	120.4	N3—C10—C9	121.7 (8)
C3—C2—H2	120.4	N3—C10—H10	119.2
C2—C3—C4	119.7 (9)	C9—C10—H10	119.2
C2—C3—H3	120.1		
			
N3—Pt1—N1—C5	46.4 (6)	C6—N2—C5—N1	−50.2 (10)
I1—Pt1—N1—C5	−127.7 (6)	C6—N2—C5—C4	128.4 (8)
N3—Pt1—N1—C1	−136.3 (6)	C3—C4—C5—N1	5.8 (12)
I1—Pt1—N1—C1	49.6 (6)	C3—C4—C5—N2	−172.7 (7)
N1—Pt1—N3—C6	−44.0 (6)	C10—N3—C6—C7	6.6 (12)
I2—Pt1—N3—C6	133.8 (5)	Pt1—N3—C6—C7	−170.9 (6)
N1—Pt1—N3—C10	138.6 (7)	C10—N3—C6—N2	−176.1 (7)
I2—Pt1—N3—C10	−43.6 (6)	Pt1—N3—C6—N2	6.4 (9)
C5—N1—C1—C2	3.4 (12)	C5—N2—C6—N3	52.8 (10)
Pt1—N1—C1—C2	−173.9 (6)	C5—N2—C6—C7	−129.9 (8)
N1—C1—C2—C3	0.9 (13)	N3—C6—C7—C8	−5.1 (13)
C1—C2—C3—C4	−1.9 (13)	N2—C6—C7—C8	177.6 (8)
C2—C3—C4—C5	−1.3 (12)	C6—C7—C8—C9	1.1 (13)
C1—N1—C5—N2	171.8 (7)	C7—C8—C9—C10	1.1 (14)
Pt1—N1—C5—N2	−10.8 (9)	C6—N3—C10—C9	−4.2 (12)
C1—N1—C5—C4	−6.7 (11)	Pt1—N3—C10—C9	173.1 (7)
Pt1—N1—C5—C4	170.7 (6)	C8—C9—C10—N3	0.5 (14)

### Hydrogen-bond geometry (Å, º)

**Table:**

*D*—H···*A*	*D*—H	H···*A*	*D*···*A*	*D*—H···*A*
N2—H2*N*···I1^i^	0.92	2.82	3.607 (7)	144

Symmetry code: (i) −*x*, −*y*+2, −*z*.
